# Effectiveness of a Participatory and Interactive Virtual Reality Intervention in Patients With Social Anxiety Disorder: Longitudinal Questionnaire Study

**DOI:** 10.2196/23024

**Published:** 2020-10-06

**Authors:** Hyun-Jin Kim, Seulki Lee, Dooyoung Jung, Ji-Won Hur, Heon-Jeong Lee, Sungkil Lee, Gerard J Kim, Chung-Yean Cho, Seungmoon Choi, Seung-Moo Lee, Chul-Hyun Cho

**Affiliations:** 1 Department of Psychiatry College of Medicine Chungnam National University Daejeon Republic of Korea; 2 Department of Psychiatry Chungnam National University Sejong Hospital Sejong Republic of Korea; 3 Department of Human Factors Engineering Ulsan National Institute of Science and Technology Ulsan Republic of Korea; 4 Department of Psychology Korea University Seoul Republic of Korea; 5 Department of Psychiatry College of Medicine Korea University Seoul Republic of Korea; 6 Department of Software Sungkyunkwan University Suwon Republic of Korea; 7 Digital Experience Laboratory Department of Computer Science and Engineering Korea University Seoul Republic of Korea; 8 Department of Film & Multimedia Korea National University of Arts Seoul Republic of Korea; 9 Department of Computer Science and Engineering Pohang University of Science and Technology Pohang Republic of Korea

**Keywords:** anxiety, social anxiety disorder, virtual reality, intervention, effectiveness, questionnaires

## Abstract

**Background:**

Social anxiety disorder (SAD) is characterized by excessive fear of negative evaluation and humiliation in social interactions and situations. Virtual reality (VR) treatment is a promising intervention option for SAD.

**Objective:**

The purpose of this study was to create a participatory and interactive VR intervention for SAD. Treatment progress, including the severity of symptoms and the cognitive and emotional aspects of SAD, was analyzed to evaluate the effectiveness of the intervention.

**Methods:**

In total, 32 individuals with SAD and 34 healthy control participants were enrolled in the study through advertisements for online bulletin boards at universities. A VR intervention was designed consisting of three stages (introduction, core, and finishing) and three difficulty levels (easy, medium, and hard) that could be selected by the participants. The core stage was the exposure intervention in which participants engaged in social situations. The effectiveness of treatment was assessed through Beck Anxiety inventory (BAI), State‐Trait Anxiety Inventory (STAI), Internalized Shame Scale (ISS), Post-Event Rumination Scale (PERS), Social Phobia Scale (SPS), Social Interaction Anxiety Scale (SIAS), Brief-Fear of Negative Evaluation Scale (BFNE), and Liebowitz Social Anxiety Scale (LSAS).

**Results:**

In the SAD group, scores on the BAI (*F*=4.616, *P*=.009), STAI-Trait (*F*=4.670, *P*=.004), ISS (*F*=6.924, *P*=.001), PERS-negative (*F*=1.008, *P*<.001), SPS (*F*=8.456, *P*<.001), BFNE (*F*=6.117, *P*=.004), KSAD (*F*=13.259, *P*<.001), and LSAS (*F*=4.103, *P*=.009) significantly improved over the treatment process. Compared with the healthy control group before treatment, the SAD group showed significantly higher scores on all scales (*P*<.001), and these significant differences persisted even after treatment (*P*<.001). In the comparison between the VR treatment responder and nonresponder subgroups, there was no significant difference across the course of the VR session.

**Conclusions:**

These findings indicated that a participatory and interactive VR intervention had a significant effect on alleviation of the clinical symptoms of SAD, confirming the usefulness of VR for the treatment of SAD. VR treatment is expected to be one of various beneficial therapeutic approaches in the future.

**Trial Registration:**

Clinical Research Information Service (CRIS) KCT0003854; https://cris.nih.go.kr/cris/search/search_result_st01.jsp?seq=13508

## Introduction

Social anxiety disorder (SAD) is characterized by excessive fear of negative evaluation and humiliation in social interactions (eg, meeting unfamiliar people) and situations (eg, being observed while eating or drinking, and performing in front of others) [1]. Individuals with SAD avoid expressing opinions, talking to people, and forming friendships with colleagues. People with high social anxiety tend to negatively interpret ambiguous social information [2]. Therefore, individuals with SAD show negative interpersonal behavior, including conflict and emotional avoidance. This weakens interpersonal relationships and ultimately leads to social isolation [3]. In particular, SAD can impair work, research, and social life [4,5], as well as reduce well-being [6].

Contemporary theories of SAD emphasize the role of cognitive processes in maintenance of the disorder [7]. Individuals with SAD feel fear and anxiety about being embarrassed in social situations. Cognitive aspects of social anxiety include recalling past experiences of failure and having postevent negative ruminations, both of which exacerbate anxiety by negatively predicting future social events [8]. In addition, individuals with SAD are very sensitive to negative evaluation and social rejection [1]. People with internalized shame are likely to experience anxiety in interpersonal relationships, as this is based on awareness of negative evaluations from others [9].

Cognitive behavior therapy (CBT) is an effective treatment that targets the characteristics of SAD. CBT supposes that when people who experience social anxiety are exposed to socially threatening situations, negative thoughts are automatically evoked, triggering unstable behaviors, emotions, and physical reactions [10,11]. CBT helps identify the unhealthy core beliefs and rigid personal rules that contribute to social anxiety, and then provides various skills and strategies to test and weaken unhealthy attitudes, and to develop and strengthen alternatives. However, people with social anxiety symptoms have high barriers to seeking help from experts due to fear of the stigma surrounding mental health [12]. Additionally, patients with SAD are often not treated as the cost of treatment is high, and it is difficult to access information about professional treatments and services available to individuals. Moreover, the waiting time for treatment is long and the treatment barrier is high due to limited access to specialized services [13]. Therefore, the accessibility of CBT via mobile and desktop computers has been increasing [14], and the scope of treatment through virtual reality (VR) has been expanding recently [15].

VR creates a virtual environment that is blocked from the outside, providing the feeling of being in a new life-sized, computer-generated environment in which one can be immersed. Stereo audio, hand controllers, and eye trackers can be used to create a much more immersive experience. Safety, cost effectiveness, and convenience are advantages to using VR in psychiatric settings. VR can provide exposure treatment to individuals in a safer way than actual exposure, allowing for a quick response and change of stimulus factors if patients experience difficulty. It also reduces the time and costs involved in real exposure treatment [15].

Research has demonstrated the effectiveness of VR for several psychiatric conditions such as anxiety disorder, eating disorder, posttraumatic stress disorder, fear of misconduct, and arachnophobia [16]. VR therapy has an advantage of alleviating the burden of exposure treatment as it is difficult to configure specific, appropriate in vivo exposure conditions for people with SAD [17]. The effectiveness of the therapy can be enhanced through participatory VR because the patients can participate directly with the controllers.

In recent VR treatment research, an environment such as a virtual street, bus, or cafe was created. The results of the research demonstrated less anxiety and paranoia about social encounters in everyday life. In addition, patients reported social interaction anxiety, reduced depressive symptoms, and improved quality of life after VR treatment [18]. In a VR treatment study that presented scenarios such as speaking in front of an audience in a conference room, interviewing, self-introduction, and talking with relatives in an apartment, this intervention was effective in alleviating symptoms of social anxiety [19]. Interactive and participatory VR therapies have the advantage of providing high-immersion situations for participants. The incorporation of interactive virtual scenarios into VR therapy might more adequately target the idiosyncratic fears of participants with SAD.

The purpose of this study was to create a participatory and interactive VR intervention for the treatment of SAD and to evaluate the effectiveness of the intervention as the treatment progressed. Most VR treatments offer a variety of scenarios to individuals with SAD, but each scenario has limitations in that they are not tailored to the patient. In this study, the VR intervention consisted of scenarios in which the participant makes a presentation and the response of others varied according to the level of difficulty. This scenario allows for the provision of patient-specific VR treatment, and is configured to be more immersive and participatory. The VR scenarios were created considering patients with SAD as the target audience. The intervention was assessed in young adults with SAD, specifically comparing the characteristic features of SAD from baseline to after several intervention sessions.

## Methods

### Participants

We recruited individuals for the SAD and healthy control groups through advertisements for online bulletin boards at universities. The inclusion criteria for the SAD group were as follows: (1) Korean-speaking men or women between the ages of 19 and 31 years, (2) met the Diagnostic and Statistical Manual of Mental Disorders-IV criteria for SAD assessed by Mini-International Neuropsychiatric Interview [20], (3) individuals who were psychotropic medication–naïve without a psychiatric comorbidity (excluding depressive disorder and panic disorder), (4) not currently receiving psychotherapy, (5) no current medical or neurological diagnoses, (6) no history of psychotic symptoms vulnerable to a VR experience, and (7) not vulnerable to visual stimuli such as epilepsy. Exclusion criteria were as follows: (1) previous history of intellectual disability or organic brain damage, (2) experienced psychotic symptoms vulnerable to VR experiences, (3) vulnerable to visual stimuli such as epilepsy, and (4) unsuitable for participation in magnetic resonance imaging research. Healthy control participants had no other neurological or psychiatric diagnoses. To determine whether the intervention had a clear, positive effect on individuals with SAD, only participants who had a score of 82 or higher on the Korean Social Avoidance and Distress Scale (KSAD) were enrolled [21]. The KSAD is a measure of the degree of experiencing anxiety in social situations and the tendency to avoid, and consists of 28 items assessed on a 5-point scale. Potential participants completed the KSAD online before enrollment. After the enrollment of participants who had a score of 82 or higher on the KSAD, we were able to collect data from a more homogeneous group of individuals with SAD who clearly had social anxiety.

**Figure 1:**
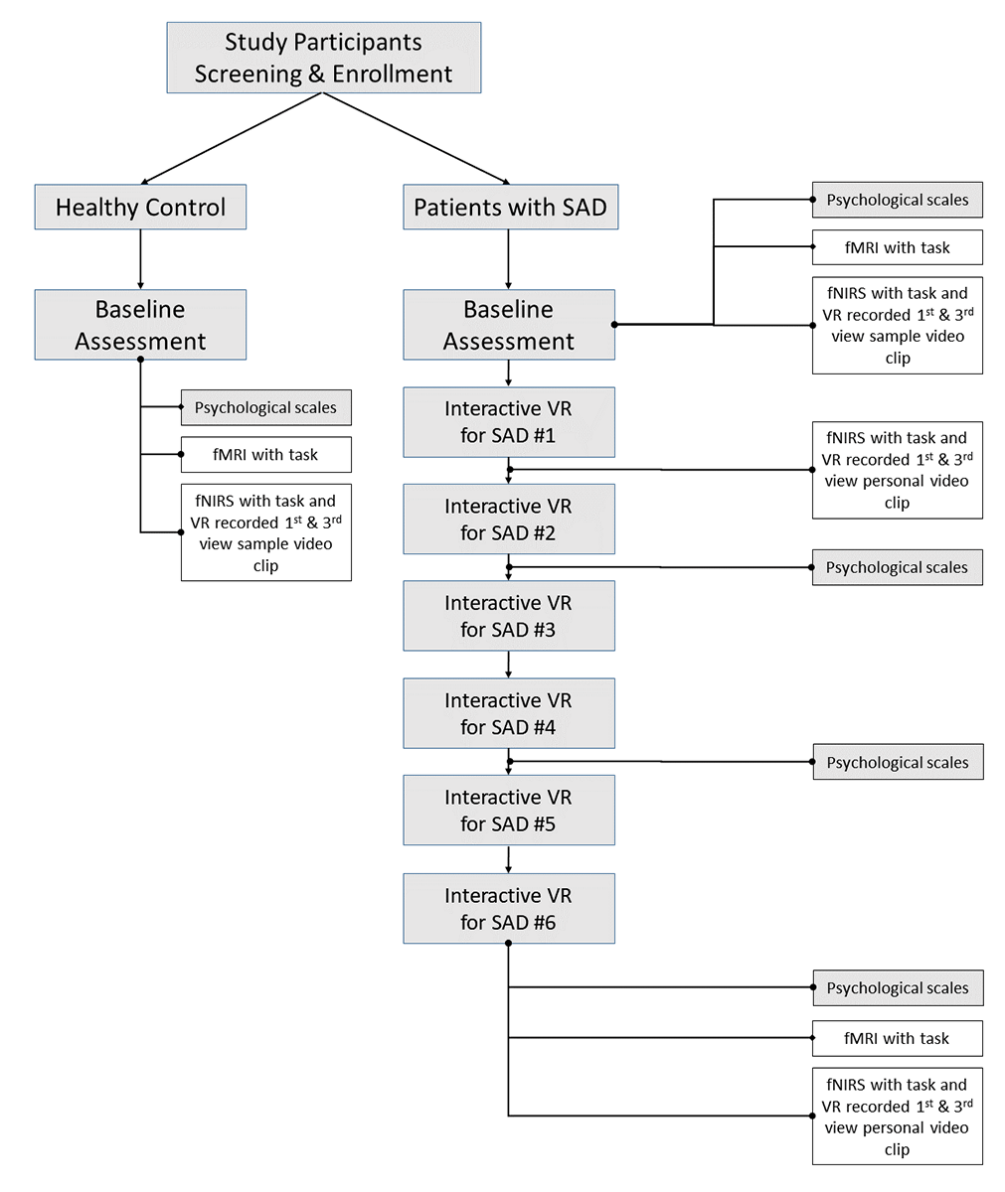
Overview of research flow chart for the entire project of participatory and interactive virtual reality (VR) treatment in patients with social anxiety disorder (SAD). This study reports the results of the analyzed data from the psychological scales. The content in the unshaded boxes represents different studies that will be presented in other papers. fMRI: functional magnetic resonance imaging; fNIRS: functional near-infrared spectroscopy.

A total of 40 patients with SAD and 34 healthy participants were enrolled in the study. Of these, 8 patients with SAD and 1 healthy control participant dropped out for personal reasons (eg, time constraints). Thus, a total of 32 patients with SAD and 33 healthy control participants completed this study. This study was part of a larger project that was conducted to evaluate the effects of interactive and participatory VR solutions using psychological scales, functional near-infrared spectroscopy, functional magnetic resonance imaging, and several physiological signals. An overview of the entire study is presented in Figure 1, and among these, only the psychological scale results were analyzed for this study to reduce distraction and clarify the subject. Other results will be presented in subsequent articles.

The study was approved by the Korea University Anam Hospital Institutional Review Board and was conducted in accordance with the Declaration of Helsinki. All participants provided informed written consent after explanation of the study procedures.

### Participatory and Interactive VR Intervention for SAD

#### Composition and Content of the VR Intervention

The participatory and interactive VR intervention for the control of social anxiety symptoms consisted of three stages (introduction, core, and finishing stages) and was divided into three levels (easy, medium, and hard) according to the difficulty of the core stage content. The VIVE (HTC Corporation, Taiwan) VR headset was used for the intervention, and the heart rate, skin tension, and eye movement of the participant during the VR experience were measured.

**Figure 2:**
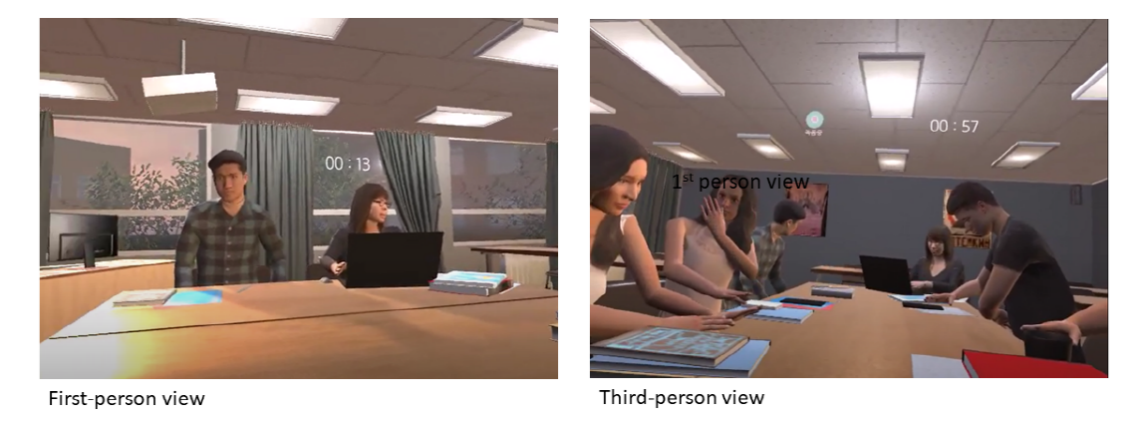
Screenshots of participatory and interactive virtual reality (VR) treatment in patients with social anxiety disorder, shown from the first- and third-person views [22].

In the introduction stage, participants were invited to select their own avatar and learn how to use the VR interface. To help participants adapt to VR and calm their mind, they partook in a meditation-based warm-up session. The core stage was the exposure intervention in which participants engaged in social situations. The situation was to enter a room where they were going to meet several other college students who were to introduce themselves to each other. In the virtual situation, 7 to 8 nonplaying characters appeared and introduced themselves (Figure 2). Once they finished their introductions, the participant with SAD pressed the record button on the screen to introduce themselves. The nonplaying characters in the scenario listened to the participant’s introduction, and the level of difficulty (easy, medium, and hard) was determined according to the nonplaying characters’ attitude and degree of reaction to the participant. As the level of difficulty increased, the attitudes of the nonplaying characters who listened to the participant’s introduction changed, in that they became more distracted and made small talk among themselves. At the hard level, one of the nonplaying characters challenged the participant when they were introducing himself/herself by saying, “Please introduce yourself properly.” The finishing stage presented general cognitive and behavioral safety guidelines for SAD in both voice and text form in VR [22].

#### Number of VR Sessions and Procedure

The VR intervention was designed for participants to perform a total of 6 sessions. Participants were allowed to perform 2 sessions in a row in a single visit, and the first session was started at an easy level. During the second session, participants could select and proceed to their desired level. It was explained that participants could stop at any time during the VR experience. Researchers were present throughout the VR experience to deal with any unexpected situations.

Participants completed a battery of assessments to evaluate the psychological state before and after the therapeutic VR sessions. Participants answered the self-reported psychological scales four times: at baseline (before the VR experience), after the second VR session, after the fourth session, and after termination (ie, after the sixth session).

### Assessments

#### Beck Anxiety Inventory

The Beck Anxiety Inventory (BAI) [23] measures the occurrence and severity of anxiety symptoms. The BAI consists of 21 items and each answer is scored on a scale of 0 (not at all) to 3 (severely). Higher total scores indicate more severe anxiety symptoms. We used the Korean version of the BAI (K-BAI) [24].

#### State-Trait Anxiety Inventory

The State-Trait Anxiety Inventory (STAI) [25] was designed to measure state and trait anxiety in research and clinical practice. State anxiety is defined as a transient, momentary emotional status that results from situational stress, whereas trait anxiety represents a predisposition to react with anxiety in stressful situations [25]. The STAI consists of two subscales for measuring the intensity of anxiety as an emotional state (20 items) and individual differences in anxiety proneness as a personality trait (20 items). Each answer is scored on a scale of 1 (not at all) to 4 (almost always/very much so). We used a Korean version of the STAI (K-STAI) [26].

#### Social Phobia Scale

The Social Phobia Scale (SPS) [27] was designed to measure the level of anxiety and fear in various social performance situations. The SPS consists of 20 items and each answer is scored on a scale of 0 (not at all) to 4 (extremely). We used the Korean version of the SPS (K-SPS) [28].

#### Social Interaction Anxiety Scale

The Social Interaction Anxiety Scale (SIAS) [27] was designed to measure fear of social interaction situations such as meeting and talking with other people. Each question is presented in the form of a self-describing statement that describes cognitive, emotional, and behavioral responses in various social interaction situations. We used the Korean-Social Interaction Anxiety Scale (K-SIAS) [28,29]. The K-SIAS consists of 20 items and each answer is scored on a scale of 0 (not at all) to 4 (extremely).

#### Brief-Fear of Negative Evaluation Scale

The Brief-Fear of Negative Evaluation Scale (BFNE) measures the fear of being negatively evaluated by other people. The original, longer scale was developed by Watson and Friend [30], while the short-form version selected only items that had a correlation of 0.50 or more with the overall score. The Korean version (K-BFNE) developed by Lee and Choi [21] was used in this study. The K-BFNE consists of 12 items and each answer is scored on a scale of 1 (strongly disagree) to 5 (strongly agree).

#### Internalized Shame Scale

The Internalized Shame Scale (ISS) [31] assesses shame proneness and internalized shame. The ISS has 30 items that consist of two basic scales for shame (24 items) and self-esteem (6 items). Shame-related questions evaluate the extent of shame that becomes magnified and internalized. We used the Korean version of the ISS (K-ISS) [32]. A factor analysis of the K-ISS identified a 4-factor structure: inadequacy (10 items), emptiness (5 items), self-punishment (5 items), and fear of mistakes (4 items).

#### Post-Event Rumination Scale

The Post-Event Rumination Scale (PERS) [33,34] was designed to measure the frequency of postevent ruminations in social situations. The PERS comprises two scales including negative rumination (15 items) and positive rumination (9 items). Each answer is scored on a scale of 0 (never) to 4 (very often); higher scores indicate more frequent rumination. We used the Korean version of the PERS (K-PERS) [35].

#### Liebowitz Social Anxiety Scale

The Liebowitz Social Anxiety Scale (LSAS) [36] assesses the degree of anxiety and avoidance in several typical social and performance situations. The LSAS consists of 24 items, which are answered for the degree of fear or anxiety (0, none to 3, severe) and avoidance (0, never to 3, usually). Higher total scores indicate more severe social anxiety symptoms. We used the Korean version of the LSAS (K-LSAS) [37].

### Statistical Analysis

To examine the difference in categorical and continuous variables between the 2 groups (SAD and healthy control groups), we used the χ^2^ test and the independent *t* test, respectively. To compare differences in the scores on the psychological scales between the SAD group and healthy control group at baseline and after termination, *t* tests were conducted. Repeated-measures analysis of variance (ANOVA) was conducted to investigate changes in the scores of the psychological scales in participants with SAD over the course of the VR intervention. All statistical analyses were performed using SPSS software version 24.0 (IBM Corp, Armonk, NY, USA). The significance level was set at *P*<.05 (two-tailed).

## Results

Demographic information showed that the mean age of the SAD (n=32) and healthy control (n=33) participants was 23.12 (SD 3.12) and 23.55 (SD 3.38) years, respectively, with no significant difference (*P*=.60). There was also no significant intergroup difference in gender distribution (*P*=.16), with 11 males (34%) and 21 females (66%) in the SAD group, and 17 males (52%) and 16 females (49%) in the healthy control group. The mean education duration of the SAD group was 14.59 years (SD 1.37) and that of the healthy control group was 14.94 years (SD 1.77), which was not significantly different (*P*=.38).

The psychological scales related to SAD were classified into several categories: general anxiety symptoms (BAI, STAI), SAD symptoms (SPS, SIAS, BFNE, KSAD, and LSAS), shame (ISS), and rumination (PERS). The comparison of scores on the psychological scales between the healthy control and SAD groups at baseline is shown in Table 1. For each scale, scores were significantly higher in the SAD group compared with those of the healthy control group (all *P*<.001).

**Table 1:** Comparison of psychological states between the social anxiety disorder (SAD) and healthy control (HC) groups at baseline.

Measures			SAD group (n=32), mean (SD)	HC group (n=33), mean (SD)	*t*	df	*P* value
BAI^a^			15.03 (10.46)	3.39 (3.89)	6.16	63	<.001
STAI-S^b^			48.72 (9.78)	35.42 (7.50)	8.45	63	<.001
STAI-T^c^			52.28 (10.50)	33.49 (7.16)	9.82	47.1	<.001
SPS^d^			30.63 (13.69)	5.76 (5.50)	9.71	48.25	<.001
SIAS^e^			42.81 (12.51)	18.30 (6.98)	7.76	63	<.001
BFNE^f^			43.63 (8.99)	27.94 (7.25)	14.71	61.85	<.001
KSAD^g^			107.34 (14.60)	49.03 (17.29)	8.73	63	<.001
LSAS^h^			72.34 (23.54)	21.85 (15.58)	9.96	53.56	<.001
**ISS^i^**							
	Total		50.09 (17.30)	16.15 (9.26)	7.84	41.82	<.001
	Inadequacy		16.34 (7.98)	4.33 (3.43)	7.32	63	<.001
	Emptiness		10.88 (4.81)	3.30 (3.34)	8.27	38.78	<.001
	Self-punishment		10.09 (5.10)	2.18 (1.84)	9.99	63	<.001
	Fear of mistake		12.78 (2.24)	6.33 (2.91)	4.69	63	<.001
**PERS^j^**							
	Negative rumination		31.38 (10.88)	9.52 (7.37)	–4.87	53.23	<.001
	Positive rumination		15.06 (6.28)	25.33 (10.29)	9.55	40.50	<.001

^a^BAI: Beck Anxiety Inventory.

^b^STAI-S: State-Trait Anxiety Inventory–State.

^c^STAI-T: Sate-Trait Anxiety Inventory–Trait.

^d^SPS: Social Phobia Scale.

^e^SIAS: Social Interaction Anxiety Scale.

^f^BFNE: Brief-Fear of Negative Evaluation Scale.

^g^KSAD: Korean Social Avoidance and Distress Scale.

^h^LSAS: Liebowitz Social Anxiety Scale.

^i^ISS: Internalized Shame Scale.

^j^PERS: Post-Event Rumination Scale.

### Changes in Social Anxiety

Repeated-measures ANOVA was conducted to assess changes in scores from baseline on the psychological scales in the participants with SAD after 2, 4, and 6 VR sessions. As shown in Table 2, general anxiety symptoms as measured by the BAI and STAI-T significantly improved after treatment, whereas the STAI-S did not improve significantly. SPS, SIAS, KSAD, BFNE, and LSAS, which are measures to evaluate the symptoms of SAD, all showed significant improvement after VR. The ISS showed significant improvement on the overall scale, and on the emptiness, self-punishment, and fear of mistakes subscales. There was no significant difference in inadequacy. Negative rumination, which is a subscale of the PERS, showed a significant improvement after the VR treatment, but positive rumination did not (Multimedia Appendix 1, Table 2).

**Table 2:** Changes in psychological states of participants with social anxiety disorder.

Measures		Baseline, mean (SD)	Session 2, mean (SD)	Session 4, mean (SD)	Session 6, mean (SD)	*F* statistic (df)	*P* value
BAI^a^		15.03 (10.46)	11.97 (9.33)	12.94 (11.29)	9.94 (9.58)	4.616 (2.348)	.009
STAI-S^b^		48.72 (9.78)	48.25 (10.14)	49.44 (11.97)	46.25 (9.96)	1.264 (2.684)	.29
STAI-T^c^		52.28 (10.50)	49.88 (11.02)	51.81 (10.97)	47.56 (11.57)	4.670 (2.556)	.004
SPS^d^		30.63 (13.69)	27.16 (13.61)	25.22 (13.70)	23.00 (13.03)	8.456 (2.400)	<.001
SIAS^e^		42.81 (12.51)	41.06 (11.86)	37.44 (12.34)	35.47 (12.83)	13.155 (2.734)	<.001
BFNE^f^		43.63 (8.99)	39.31 (11.00)	38.75 (7.72)	37.59 (7.29)	6.117 (2.027)	.004
KSAD^g^		107.34 (14.60)	104.16 (14.28)	99.66 (13.37)	97.69 (12.55)	13.259 (2.369)	<.001
LSAS^h^		71.34 (4.16)	74.03 (4.77)	68.28 (4.13)	64.00 (4.11)	4.103 (2.503)	.009
**ISS** ^i^							
	Overall	50.09 (17.30)	43.91 (17.40)	43.13 (18.34)	38.78 (16.85)	6.924 (2.372)	.001
	Inadequacy	16.34 (7.98)	14.75 (8.38)	14.72 (8.22)	13.44 (7.81)	2.604 (2.719)	.06
	Emptiness	10.88 (4.81)	8.81 (5.52)	8.81 (5.03)	7.69 (4.47)	5.152 (2.769)	.002
	Self-punishment	10.09 (5.09)	9.16 (4.68)	8.41 (4.76)	7.50 (4.33)	5.528 (2.194)	.005
	Fear of mistake	12.78 (2.24)	11.19 (2.42)	11.25 (3.20)	10.16 (3.05)	10.891 (2.662)	<.001
**PERS** ^j^							
	Negative rumination	31.38 (10.88)	27.03 (10.97)	25.81 (10.78)	23.41 (11.10)	6.974 (2.730)	<.001
	Positive rumination	15.06 (6.29)	13.13 (5.84)	14.47 (6.73)	13.56 (6.83)	1.008 (2.812)	.39

^a^BAI: Beck Anxiety Inventory.

^b^STAI-S: State-Trait Anxiety Inventory–State.

^c^STAI-T: Sate-Trait Anxiety Inventory–Trait.

^d^SPS: Social Phobia Scale.

^e^SIAS: Social Interaction Anxiety Scale.

^f^BFNE: Brief-Fear of Negative Evaluation Scale.

^g^KSAD: Korean Social Avoidance and Distress Scale.

^h^LSAS: Liebowitz Social Anxiety Scale.

^i^ISS: Internalized Shame Scale.

^j^PERS: Post-Event Rumination Scale.

Posthoc analyses for pairwise comparisons between baseline to sessions 2, 4, and 6 were performed by the Bonferroni method. The scales of BAI (*P*=.003), and overall (*P*=.01), emptiness (*P*=.001), and self-punishment (*P*=.001) of the ISS showed significant differences between baseline and session 6. The scales of PERS (*P*=.01), SPS (*P*=.005), SIAS (*P*<.001), BFNE (*P*=.01), and KSAD (*P*<.001) showed significant differences between baseline and session 4. The fear mistake subscale of the ISS (*P*=.005) showed a significant difference between baseline and session 2 (Multimedia Appendix 1).

### Changes in Psychological States Between Responder and Nonresponder Subgroups After VR Treatment

In general, the response to treatment is defined as a reduction of 50% or more from the baseline psychological scale. We conducted a repeated-measures ANOVA for comparative analysis by dividing the participants into subgroups: those with a 50% or more reduction of the BAI scale value compared to the baseline were defined as the treatment responder group, and those with a less than 50% reduction were classified as the nonresponder group.

As presented in Table 3, all psychological scale values except for STAI-S, positive subscale of the PERS, and inappropriate subscale of the ISS showed significant changes across the VR treatment session in both subgroups. A significant session-by-group interaction was found for the BAI value; however, no session-by-group interaction was observed for the other psychological scales.

**Table 3:** Changes in psychological states between responder and nonresponder subgroups according to virtual reality treatment.^a^

Measures		Responders (n=14), mean (SD)		Nonresponders (n=18)*,* mean (SD)	Main effect of session		Session-by-group interaction		
					*F* (df)	*P* value		*F* (df)	*P* value
**BAI^b^**					6.021 (2.446)	.002		4.684 (2.446)	.008
	Baseline	12.93 (10.28)		16.68 (10.60)					
	Session 2	8.71 (7.79)		14.50 (9.84)					
	Session 4	6.57 (6.05)		17.89 (12.04)					
	Session 6	3.21 (4.04)		15.17 (9.41)					
**STAI-S^c^**					1.178 (2.737)	.32		1.087 (2.737)	.36
	Baseline	45.64 (8.39)		51.11 (10.32)					
	Session 2	44.79 (8.53)		50.94 (10.70)					
	Session 4	43.14 (6.21)		54.33 (13.18)					
	Session 6	41.93 (6.22)		49.61 (11.14)					
**STAI-T^d^**					4.875 (2.515)	.006		1.675 (2.515)	.19
	Baseline	47.00 (10.06)		56.39 (9.10)					
	Session 2	46.00 (10.68)		52.89 (10.58)					
	Session 4	57.06 (9.04)		57.06 (9.50)					
	Session 6	40.57 (8.93)		53.00 (10.56)					
**SPS^e^**					8.849 (2.407)	<.001		0.983 (2.407)	.39
	Baseline	26.00 (15.65)		34.22 (11.11)					
	Session 2	23.14 (15.75)		30.28 (11.16)					
	Session 4	18.64 (13.18)		30.33 (12.18)					
	Session 6	16.71 (12.39)		27.89 (11.58)					
**SIAS^f^**					13.819 (2.659)	<.001		1.418 (2.659)	.25
	Baseline	37.21 (12.21)		47.17 (11.21)					
	Session 2	36.43 (11.79)		44.67 (10.91)					
	Session 4	29.86 (11.55)		43.33 (9.56)					
	Session 6	29.21 (10.37)		40.33 (12.67)					
**BFNE^g^**					5.976 (2.052)	.004		0.672 (2.052)	.52
	Baseline	41.93 (9.56)		44.94 (8.56)					
	Session 2	39.36 (9.96)		39.28 (12.04)					
	Session 4	36.64 (7.89)		40.39 (7.39)					
	Session 6	35.64 (6.71)		39.11 (7.55)					
**KSAD^h^**					12.854 (2.359)	<.001		0.168 (2.359)	.88
	Baseline	102.36 (13.68)		111.22 (14.46)					
	Session 2	97.93 (8.57)		109.00 (16.09)					
	Session 4	94.29 (12.30)		103.83 (12.95)					
	Session 6	91.71 (5.89)		102.33 (14.44)					
**LSAS^i^**					3.024 (2.471)	.04		0.727 (2.471)	.51
	Baseline	33.50 (12.73)		42.67 (11.51)					
	Session 2	36.29 (15.51)		43.11 (10.42)					
	Session 4	31.07 (11.10)		42.72 (10.38)					
	Session 6	29.29 (9.43)		39.94 (11.02)					
**ISS^j^ Overall**					6.835 (2.345)	.001		0.309 (2.345)	.77
	Baseline	45.79 (18.97)		53.44 (15.60)					
	Session 2	40.86 (18.22)		46.28 (16.87)					
	Session 4	39.07 (18.12)		46.28 (18.38)					
	Session 6	33.00 (15.49)		43.28 (16.89)					
**ISS Inadequacy**					2.574 (2.698)	.07		0.343 (2.698)	.77
	Baseline	14.36 (8.42)		17.89 (7.50)					
	Session 2	13.57 (8.65)		15.67 (8.29)					
	Session 4	12.86 (8.03)		16.17 (8.30)					
	Session 6	11.07 (6.96)		15.28 (8.12)					
**ISS Emptiness**					5.090 (2.761)	.004		0.350 (2.761)	.77
	Baseline	9.86 (5.11)		11.67 (4.55)					
	Session 2	8.14 (4.96)		9.33 (6.01)					
	Session 4	8.07 (5.03)		9.39 (5.09)					
	Session 6	6.14 (3.44)		8.89 (4.89)					
**ISS - Self-punishment**					5.486 (2.198)	.005		0.143 (2.198)	.88
	Baseline	9.43 (5.12)		10.61 (5.16)					
	Session 2	8.21 (4.71)		9.89 (4.65)					
	Session 4	7.29 (4.43)		9.28 (4.96)					
	Session 6	6.43 (4.43)		8.33 (4.17)					
**ISS Fear of mistake**					10.598 (2.645)	<.001		0.417 (2.645)	.72
	Baseline	12.14 (2.45)		13.28 (1.99)					
	Session 2	10.93 (2.76)		11.39 (2.17)					
	Session 4	10.86 (2.93)		11.56 (3.45)					
	Session 6	9.36 (3.32)		10.78 (2.76)					
**PERS^k^ Negative rumination**					6.931 (2.720)	.001		0.354 (2.720)	.77
	Baseline	28.14 (10.31)		33.89 (10.93)					
	Session 2	24.71 (12.64)		28.83 (9.45)					
	Session 4	22.29 (12.00)		28.56 (9.14)					
	Session 6	19.00 (8.69)		26.83 (11.77)					
**PERS Positive rumination**					0.855 (2.822)	.46		0.375 (2.822)	.76
	Baseline	15.29 (5.01)		14.89 (7.27)					
	Session 2	14.43 (5.69)		12.11 (5.90)					
	Session 4	15.64 (6.46)		13.56 (6.97)					
	Session 6	15.21 (6.80)		12.28 (6.76)					

^a^Treatment responder and nonresponder subgroups were divided according to the reduction of the BAI scale value by 50% or more compared to the baseline value.

^b^BAI: Beck Anxiety Inventory.

^c^STAI-S: State-Trait Anxiety Inventory–State.

^d^STAI-T: Sate-Trait Anxiety Inventory–Trait.

^e^SPS: Social Phobia Scale.

^f^SIAS: Social Interaction Anxiety Scale.

^g^BFNE: Brief-Fear of Negative Evaluation Scale.

^h^KSAD: Korean Social Avoidance and Distress Scale.

^i^LSAS: Liebowitz Social Anxiety Scale.

^j^ISS: Internalized Shame Scale.

^k^PERS: Post-Event Rumination Scale.

### Comparison of SAD and Control Groups After VR Treatment

We analyzed whether the scores on the psychological scales differed in the SAD group compared with those of the healthy control group after 6 VR treatment sessions. Results from independent *t* tests showed that even after completing the VR sessions, the SAD group continued to have significantly higher scores than the healthy control group on all psychological scales (Table 4).

**Table 4:** Comparison of psychological states between the social anxiety disorder (SAD) and healthy control (HC) groups after virtual reality treatment.

Measures		SAD group (n=32), mean (SD)	HC group^a^ (n=33), mean (SD)	*t*	df	*P* value
BAI^b^		9.94 (9.58)	3.39 (3.89)	3.59	40.70	.001
STAI-S^c^		46.25 (46.25)	35.42 (35.42)	4.96	63	<.001
STAI-T^d^		47.56 (11.57)	33.49 (7.16)	5.92	63	<.001
SPS^e^		23.00 (13.03)	5.76 (5.50)	6.91	41.45	<.001
SIAS^f^		35.47 (12.83)	18.30 (6.98)	6.67	47.54	<.001
BFNE^g^		37.59 (7.29)	27.94 (7.25)	5.35	63	<.001
KSAD^h^		97.69 (12.55)	49.03 (17.29)	13.01	58.43	<.001
LSAS^i^		64.00 (23.22)	21.85 (15.58)	8.62	63	<.001
**ISS^j^**						
	Overall	38.78 (16.85)	16.15 (9.26)	6.69	47.85	<.001
	Inadequacy	13.43 (7.81)	4.33 (3.43)	6.05	42.28	<.001
	Emptiness	7.69 (4.47)	3.30 (3.43)	4.45	63	<.001
	Self-punishment	7.50 (4.33)	2.18 (1.84)	6.41	41.65	<.001
	Fear of mistake	10.16 (3.05)	6.33 (2.91)	5.17	63	<.001
**PERS^k^**						
	Negative rumination	23.41 (11.10)	9.52 (7.37)	5.92	53.66	<.001
	Positive rumination	13.56 (6.82)	25.33 (10.29)	-5.45	55.76	<.001

^a^Since the control group did not receive the virtual reality treatment, psychological states of the healthy control group were measured only at baseline.

^b^BAI: Beck Anxiety Inventory.

^c^STAI-S: State-Trait Anxiety Inventory–State.

^d^STAI-T: Sate-Trait Anxiety Inventory–Trait.

^e^SPS: Social Phobia Scale.

^f^SIAS: Social Interaction Anxiety Scale.

^g^BFNE: Brief-Fear of Negative Evaluation Scale.

^h^KSAD: Korean Social Avoidance and Distress Scale.

^i^LSAS: Liebowitz Social Anxiety Scale.

^j^ISS: Internalized Shame Scale.

^k^PERS: Post-Event Rumination Scale.

## Discussion

### Principal Findings

The aim of this study was to analyze the effectiveness of a newly developed participatory and interactive VR intervention for individuals with SAD. In particular, using psychological tests related to SAD, we evaluated whether the VR intervention was effective in improving various symptoms.

General anxiety symptoms, measured by the BAI and STAI-T, were significantly improved compared with those measured before treatment, whereas state anxiety (ie, STAI-S) was not. State anxiety increases the threat value assigned to a stimulus or situation, and trait anxiety gives rise to a tendency to constantly direct attention toward the source of threat [38]. Any trait that does not change easily over a lifetime is a distinguishing feature of a person’s character, and anxiety-related traits are among the most important risk factors of anxiety disorder [39].

The scales that directly assess the symptoms of SAD showed significant improvement compared with those measured before treatment. SAD can be divided into symptoms that appear in specific situations and general social interactions [27], which were assessed with the SPS and SIAS, both of which showed significant improvement after VR. Scores on the LSAS and KSAD were significantly reduced after treatment. However, the LSAS score increased after session 2, and then decreased in the subsequent two time points (session 4 and session 6). BAI scores decreased in session 2, and then increased in session 4 before decreasing again in session 6. Similarly, the STAI (both state and trait scales) showed a decrease from baseline to session 2, followed by an increase after session 4 and then a decrease after session 6. Although this is only a pattern, it could be speculated that the tendency of anxiety to increase during a VR session is probably due to exposure to a VR session at a stage in the middle of the intervention process before finally showing a significant therapeutic effect. This is an important consideration, because increased anxiety during a VR session may lead to a problem of dropping out of treatment due to poor treatment adherence after the initial treatment session. Treatment compliance is important in CBT because it requires a certain period of treatment, and exposure techniques can exacerbate symptoms in some cases [40]. In general, VR treatment is considered to have higher compliance than conventional treatment [41], but it is necessary to pay attention to changes in compliance during the treatment sessions.

Cognitive and emotional components are known to be involved in the development of SAD [42]. Scores on the ISS, which is used to evaluate internalized shame, improved significantly as VR treatment progressed, including a significant decrease on almost every ISS subscale (ie, emptiness, self-punishment, and fear of mistakes). Shame is an emotional component related to social anxiety that is based on a negative evaluation of the awareness of others, suggesting a positive relationship between social anxiety and shame [43-45]. A key part of cognitive models of social anxiety is that individuals perceive a negative evaluation by others [7]. In this study, negative rumination significantly improved after treatment, whereas positive rumination was not significantly different. Past studies of rumination have shown a relationship between negative rumination and social anxiety, but the relationship between social anxiety and positive rumination has been inconsistent or was not significant [33,46]. The improvement of negative rumination suggests that this can affect the cognitive structure of SAD.

Posthoc analysis to compare baseline scores to each session time point showed that VR had a significant effect on the fear of mistake subscale of the ISS at the initial point of treatment (after session 2). In comparison, BAI and the overall, emptiness, and self-punishment subscales of the ISS changed significantly only at the end of treatment (after session 6), and PERS, SPS, SIAS, BFNE, and KSAD scales showed significant changes around the middle point of VR treatment (after session 4). We could speculate that the improvement patterns of psychological symptoms may differ across the process of the VR treatment sessions. Since prolonging or shortening the number of VR treatment sessions might affect the therapeutic outcome on each psychological state, future studies are needed to determine how and when psychological symptoms change according to the VR treatment process and to establish the optimal number for VR treatment sessions.

Although we found significant improvements in SAD symptoms after treatment, these symptoms were still significantly different compared with those of the control group. This result shows that the VR intervention had a significant effect in the SAD group, but that the participants with SAD still exhibited prominent symptoms after VR treatment sessions. This suggests that the treatment effect of VR alone might not be sufficient to draw out the treatment response or remission. A meta-analysis of VR treatment focused on anxiety disorders found a small effect size [47]. Therefore, it is necessary to consider changes in treatment methods such as the combination of various treatment techniques, including conventional psychiatric treatment or more repeated VR trials, to achieve a sufficient therapeutic effect in the real world.

We did not find any significant session-by-group interaction in the majority of the psychological scales except for BAI between the two subgroups of responders and nonresponders (according to changes in the BAI scale). This result can be presumed to be due to the limitation of the methodology in which the SAD group was divided into responder and nonresponder groups according to a single psychological scale. It is challenging to determine a clear cutoff as to what scale or criteria to use for distinguishing responders. Moreover, since this study attempted to collect data from a more homogeneous sample of individuals with social anxiety symptoms (KSAD≥82), subgroup differences might not be expected. We hope to be able to derive meaningful results through further studies by comparison between subgroups according to the degree of severity based on the scores of different psychological scales, or by a comparative study according to whether or not the individual is also under medication for anxiety.

The results of this study indicated improvements in almost all elements of social anxiety measured, including general anxiety, social anxiety symptoms, and cognitive and emotional aspects of social anxiety, in the SAD group after treatment. Previous studies related to VR treatment for social anxiety have also shown an improvement in symptoms [47-49]; however, this study demonstrated an effect not only on social anxiety symptoms but also on a wide range of related factors, including general anxiety symptoms and cognitive and emotional characteristics.

### Limitations and Strengths

Our results should be interpreted within the context of the study’s limitations. First, this study did not have a sham or waitlist control group, which limits interpretation of the results. Second, this study employed self-rated scales that could be confounded by bias (eg, participant motivation). Nonetheless, a comprehensive evaluation related to social anxiety disorder was attempted in analyzing the effectiveness of treatment, including changes during treatment sessions. This study enrolled participants in the patient group who met strict diagnostic criteria and had not received any other treatments, including psychiatric drugs. Given these strengths, the therapeutic effect of a VR intervention could be evaluated more accurately.

### Conclusion

This study investigated the effectiveness of a newly developed participatory and interactive VR intervention in patients with SAD. The results show that a VR intervention can be an effective treatment for various dimensions of SAD. Technology-based treatment in psychiatry is more cost-effective, easier to handle, and more manageable for both the therapist and client. VR treatment is a promising tool in the field of psychiatry, which can simulate situations for patients with anxiety in a safe, controllable, and reproducible way [15,50,51]. Future research should focus on ensuring that the effectiveness of these immersive VR treatments persist after treatment, and that treatments are made more effective through different treatment combinations or changes in techniques.

## Appendix

Multimedia Appendix 1 Changes in psychological state due to participatory and interactive virtual reality (VR) treatment in patients with social anxiety disorder.

## Abbreviations

ANOVA: analysis of variance
BAI: Beck Anxiety Inventory
BFNE: Brief-Fear of Negative Evaluation Scale
CBT: cognitive behavioral therapy
ISS: Internalized Shame Scale
KSAD: Korean Social Avoidance and Distress Scale
LSAS: Liebowitz Social Anxiety Scale
PERS: Post-Event Rumination Scale
SAD: social anxiety disorder
SIAS: Social Interaction Anxiety Scale
SPS: Social Phobia Scale
STAI: State-Trait Anxiety Inventory
VR: virtual reality
