# Supplementary figures and images for: Effectiveness of a Participatory and Interactive Virtual Reality Intervention in Patients With Social Anxiety Disorder: Longitudinal Questionnaire Study
Source: J Med Internet Res. 2020 Oct 6;22(10):e23024. doi: 10.2196/23024 (PMC7576535; doi:10.2196/23024)

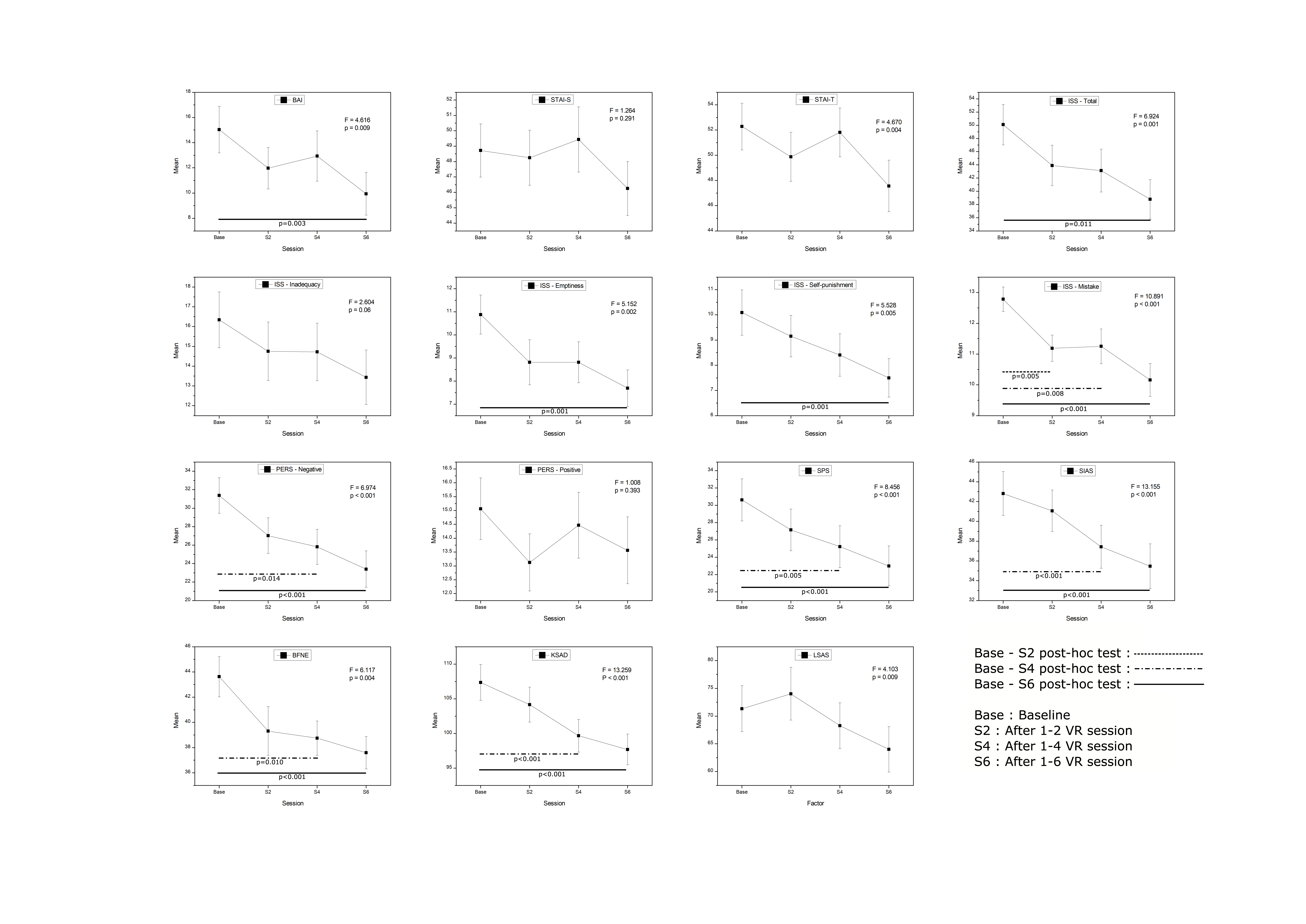

Supplement: Multimedia Appendix 1 [file jmir_v22i10e23024_app1.png]
